# Unraveling the blood transcriptome after real-life exposure of Wistar-rats to PM2.5, PM1 and water-soluble metals in the ambient air

**DOI:** 10.1016/j.toxrep.2020.10.014

**Published:** 2020-10-21

**Authors:** Ilias S. Frydas, Marianthi Kermenidou, Olga Tsave, Athanasios Salifoglou, Dimosthenis A. Sarigiannis

**Affiliations:** a Environmental Engineering Laboratory, Department of Chemical Engineering, Aristotle University of Thessaloniki, Greece; b HERACLES Research Center on the Exposome and Health – Center for Interdisciplinary Research and Innovation, Aristotle University of Thessaloniki, Greece; c Inorganic Chemistry Laboratory, Department of Chemical Engineering, Aristotle University of Thessaloniki, Greece; d Environmental Health Engineering Chair, Science, Technology and Society Department, University School for Advanced Study (IUSS), Pavia, Italy

**Keywords:** PM1, PM2.5, Microarrays, Exposure, Real-life, AOPs

## Abstract

Exposure to particulate matter (PM) is one of the most important environmental issues in Europe with major health impact. Various sizes of PM are suspended in the atmosphere and contributes to ambient air pollution. The current study aimed to explore the differential gene expression in blood, and the effect on the respective biological signaling pathways in Wistar rats, after exposure to PM2.5 and PM1 ambient air particles for an eight-week period. A control group was included with animals breathing non-filtered atmospheric air. In parallel, filtered PM2.5 and PM1 was collected in separate samplers. The results after whole genome microarray analysis showed 23 differentially expressed genes (DEGs) between control and PM2.5 group. In addition, pairwise comparison between control and PM1 group displayed 5635 DEGs linked to 69 biological pathways involved in inflammatory response, cell cycle and carcinogenicity. The smaller the size of the inhaled particles, the more gene alterations are triggered compared to non-filtered air group. More specifically, in inflammation signaling procedures differentially regulated gene expression was shown for interleukin-4 (IL-4), IL-7, IL-1, IL-5, IL-9, IL-6 and IL-2. We have identified that RASGFR1, TRIM65, TRIM33, PLEKHB1, CAR4, S100A8, S100A9, ALPL, NP4 and the PROK2 genes are potential targets for the development of adverse outcome pathways (AOPs) due to “real-life” exposure of Wistar rats. Particle measurements during the exposure period showed elevated concentrations of Fe, Mn and Zn in both PM1 and PM2.5 filter fractions, and of Cu in PM2.5. In addition, water-soluble concentration of metals showed significant differences between PM1 and PM2.5 fractions for V, Zn, As, Pb and Mn. In summary, in this study specific gene biomarkers of exposure to ambient air have been identified and heavy metals that are possibly linked to their altered regulation have been found. The results of this research will pave the way for the development of novel AOPs concerning the health effects of the environmental pollution.

## Highlights

• Development of a “real-life” exposure system to ambient PM1 and PM2.5 particles for Wistar rats.
• Blood transcriptome analysis identified differentially expressed genes as candidate biomarkers in PM1 and PM2.5 groups.
• Pathway analysis revealed differentially regulated gene expression in inflammation signaling.
• Identification of candidate metals for possible correlation with the identified candidate genes leading to the development of AOPs.

## Graphical abstract

**Figure:**
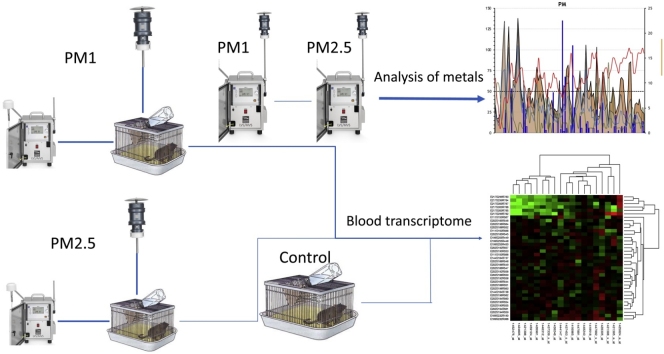

## 1 Introduction

Urban air pollution poses severe environmental and public health problems in modern industrial societies. It is known to induce short- and long-term effects on human health such as COPD (Chronic Obstructive Pulmonary Disease), cough, shortness of breath, asthma, respiratory disease, high rates of hospitalization, chronic asthma, pulmonary insufficiency, cardiovascular diseases, and cardiovascular mortality [1,2]. The link between long-term exposure to particulate air pollution smaller than 2.5 μm (PM2.5) and deaths from lung cancer and heart disease in the USA has been already shown in an earlier study, which demonstrated that each 10 μg/m^3^ rise in long-term average PM2.5 concentration is associated with an 8% increase in lung cancer mortality [3]. Particle matter size is divided in five categories (1 nm to 100 μm) depending the depth of penetration into the lung compartments [59]. Several studies have shown that the size significantly determines how deep the particles can penetrate into the lung compartments. Particles with diameters between 2.5 and 10 μm (usually defined as PM2.5 and PM10) deposit mainly in the upper airways and can be cleared by the mucociliary system, where a complex mucus barrier is located, lining the mucosal epithelium of tissues. Epidemiologic studies have observed associations between short-term increases in ambient particulate matter (PM) concentrations and increases in respiratory morbidity [4]. Particle size less than 1 μm can reach to the alveoli and the bronchioles causing bronchial inflammation and accumulation of inflammatory cells [4]. Thus, over the last few years research has focused on the evaluation of the health effects caused by the exposure not only to PM10 and PM2.5 but also PM1, an area that has been poorly investigated before, and it is possible to cause the more severe alterations on biochemical pathways.

Particulate matter toxicity is a combination of the effect caused from particles, adsorbed toxic pollutants, biological components such as endotoxin, pollen, fungal spores, viruses, and bacteria, polycyclic aromatic hydrocarbons (PAHs), volatile organic compounds (VOCs) and heavy metals, and furthermore the most challenging issue is to identify and quantify the different influences of each different chemical, physical or biological component ([5,6]). The main portal of entry for particle matter is the oral and the nasal cavity and the first protective barrier is the airway epithelium that contains a protective mucus layer [7]. The thickness of this layer is different according to the part of the respiratory system the epithelium is located. In the nasal cavity the airway epithelium has a thickness of 5–15 μm, in trachea 10–30 μm, and 2–5 μm in the bronchioles [8]. Whole body exposure is likely to result in oral uptake via grooming in addition to inhalation exposure, whereas the gastrointestinal epithelium also functions as a barrier against pathogens and environmental factors and in rats has a mean thickness of 100–300 μm in the stomach and 100–900 μm in the intestine [9,10]. Moreover, studies have shown the impact of exposure to particulate matter on the brain, showing differential gene expression and differentiation of neural biomarkers [11,12].

In environmental science PM10 and PM2.5 are not the only metrics of particle matter, and this is because PM1 and ultrafine particle matter (<0.1 μm, UFP) also contribute to particle number concentration (Morawska et al. 1999). Still, there are much less data and epidemiological evidence for PM1 and UFP, mainly because there is the assumption that when regulating for PM2.5, UFP and PM1 are also regulated (Tobias et al. 2018). Thus, currently there is an urgent need for data on the health effects of PM1 and UFP, since there are contradictory claims within the scientific community, leading to no clear answer whether the short- and long-term consequences of PM2.5 and smaller particle size are the same or not [13]. Thus, we sought to study the real-life exposure of Wistar rats in an urban environmental pollution station in the city of Thessaloniki. Studies that have been performed in cities of the USA and Germany have shown increased cardiovascular risk from exposure to UFP and not PM2.5 [14,15]. Before these studies, yet another study performed for the city of London had shown the difference on the health effects between the two particle matter categories, where cardiovascular deaths were associated to UFP and PM2.5 to respiratory health outcomes [16]. In addition, in industrial areas it is known that the PM1/PM10 ratio is higher than the one that is measured within urban environments, and because of these conclusions, research on health effects has shifted from PM10 and PM2.5 to PM1 and smaller, even though air quality standards related to the latter particle sizes are still nonexistent [17,18].

Although human exposure to fine particles has been found to be associated with biomarkers, the direct correlation between exposure and adverse health outcomes remains ambiguous and so far no research model was sufficient enough to lead to reliable conclusions [13]. The main reasons include the lack of data on the effect of fine particles on biomarkers, and the complexity of the interactions between human and environmental exposure. There is thus a need to develop a "map", which records and discloses cellular signaling pathways affected by exposure to fine particles at the genome level.

In order to understand the biological mechanisms triggered on the gene expression level by PM2.5 and PM1 exposure and lead to airway inflammation, in this study, we aimed to evaluate the following hypotheses:

(1) How the natural exposure to PM2.5 and PM1 in Wistar rats can alter the gene expression in blood after whole genome analysis using microarray probes in comparison to the control group;

(2) Which biochemical pathways are differentially regulated after exposure to different air particulate matter and what is the type of immune response triggered after exposure to PM2.5 and PM1; and

(3) How altered gene expression in rats after exposure to PM2.5 and PM1 from a traffic-related area would correlate with the concentrations of other measured components such as heavy metals.

## 2 Material and methods

### 2.1 Animal model protocol

Nine, ten-week old pathogen free male Wistar rats were randomly chosen from the same breeding batch and provided by the Laboratory of Anatomy, Histology and Embryology of the Veterinary Medicine department of the Aristotle University of Thessaloniki, weighting approximately 320–330 g. The animals were exposed for 8 weeks (24 h per day/7 days per week) in a period within January and March 2020. All animal procedures were carried out in accordance with the 2010/63/EU Directive concerning animal experiments. Animals were housed in a specific pathogen-free environment at an air pollution monitoring Isobox station at an urban site area of Thessaloniki. The ambient air measurement site is located in the Kalamaria district of eastern Thessaloniki (22° 57′ 33′.49, 40° 34′ 44′.10, 60 m altitude) a densely populated area where elevated buildings do not favor pollutants dispersion. The site is crossed by the main ring road of Thessaloniki, at a distance of approximately 500 m.

In order to adjust real-life exposure conditions a previous protocol developed for mice [19] was adapted. Briefly, three whole body chambers were constructed from plexiglass for Wistar rats exposure (90cm × 55cm x 35 cm), and they were connected to TECORA pumps equipped with an inertial impactor with cut off size at 2.5 and 1 μm respectively to remove particles larger than the reference size from the air flow, providing filtered air continuously 24 h/day, whereas to the control chamber non-filtered atmospheric air has been provided. Pump inlets distributing the filtered air to the chambers were placed on the stations’ roof three meters above the ground. The flow rates within the chambers were adjusted to 2.3 m^3^ h**^−^**^1^, and were kept stable through the experimental period. Within the chambers the rat cages were placed after keeping the animals for one week at the monitoring station for acclimatization. The airflow rates, the temperature, and the humidity were measured daily inside the chambers to ensure animal well-being and stress avoidance. Within the monitoring station the temperature was maintained at 19° to 24 °C, and the humidity at 50–60%. Light cycles followed natural light, and the animals were provided water and food ad libitum. The straw used for the rat cages was pathogen and dust-free specific for laboratory rodent animals (Viozois S.A., Greece). In addition, all the TECORA pumps were connected to an uninterruptable power supply (UPS) system in case of a power cut, and in the latter case a power failure alert system was connected and was able to send direct messages to the researchers when a power failure was ongoing. The animals were randomized into three groups: PM1-exposure, PM2.5-exposure, and control group (N = 3/group). In order to circumvent potentially confounding factors such as oestrus cycles and the impact of hormones on physiological functions (and especially thermoregulatory functions), only male rats were included in this study.

In order to monitor the PM concentrations within the chambers a light scattering laser photometer Mini Laser Aerosol Spectrometer (Mini-LAS) 11-R, is a light scattering optical sensor, developed by Grimm. The 11R measures PM1, PM2.5, PM10 and particle counts ranging from 0.25 to 32 μm and classifies it into 31 size channels. The spectrometer was calibrated according to the EN 12,341 European Standard to demonstrate reference equivalence. During the exposure period particle chamber measurements were in agreement to the air particle measurements that were performed in parallel with the animal exposure.

### 2.2 Ambient air particulate matter sampling

PM2.5 and PM1 size fractions were measured to determine the chemical composition of urban aerosols from November 2019 to mid-March of 2020 at the same air pollution monitoring station where the animals were exposed in Kalamaria, an urban area of Thessaloniki. Samplers were placed at a height of approximately 3 m from the ground. PM2.5 and PM1 samples were collected using low air flow samplers (ENCO PM, TCR TECORA, Italy). The used sampling heads meet the EN 14,907 standard and operated at a flow-rate of 38.3 L/min, with a collection time of 24 h per sample. Samples were collected on PTFE membranes filters with PMP supporting ring (PALL Life Sciences, Ø 47 mm, pore size 2 um, USA). PM mass concentrations were calculated by weighing the filters before and after sampling. Mean ambient temperature was 9.1 °C while average relative humidity was measured at 69 %.

### 2.3 Chemical analysis of PM filters

Each filter was weighed after sampling and then was cut into two equally-sized pieces using a ceramic blade in preparation for chemical analysis. The first piece of the filter was used to determine metals by using an inductively coupled plasma mass spectrometer (ICP-MS). To determine the water-soluble fraction, samples were sonicated in 10 mL of ultrapure water at room temperature for 30 min. After the extraction, the solution was filtered and acidified to 2% HNO_3_ to prevent metal adsorption. All reagents used for the digestion procedures were of ICP-MS grade (HNO_3_-Suprapur 69 %, Merck) and all solutions were prepared using ultrapure water LC–MS grade. All glassware was soaked in 6 N HNO_3_ for at least 24 h, and rinsed repeatedly with ultrapure water before use. Field blank filters were also collected and used to correct the background concentrations or influences from handling and transport. Laboratory and field blanks were extracted and analyzed in the same way as the samples. Determined limits of detection (LOD) for elements were calculated based on three times the standard deviation (3σ) of the blank values (n = 10), and ranged between 0.002 and 0.1 μg/L. All samples were analyzed using Inductive Coupled Plasma Mass Spectrometry (ICP-MS) (Thermo Icap Qc with ESI 4D autosampler). Analysis was performed by applying collision cell mode (kinetic energy discrimination (KED), using He to selectively attenuate all polyatomic interferences based on their size. The instrument used Ni sample and skimmer cones. Prior to the analysis of the samples, the ICP-MS system was allowed to equilibrate for 30 min and then the sensitivity and the stability of the instrument were checked in KED mode by using tune solution containing 1 μg/L (each) of Ba, Bi, Ce, Co, In, Li, and U in 2% HNO_3_ and 0,5% HCl. Then, a performance test in KED mode was performed using the same tune solution. When necessary auto tune and calibration mass tests were also performed to optimize the instrument operation.

### 2.4 RNA extraction and integrity assessment

Blood was retrieved from the heart of the Wistar rats and a total volume of 7.5 mL was collected in PAXgene Blood RNA tubes (Qiagen Sciences). Samples remained at least two hours at room temperature, and subsequently RNA was extracted using the RNeasy Mini Kit (Qiagen Sciences) according to manufacturer instructions and yields were measured using a NanoDrop 2000 spectrometer (ThermoScientific), and sample integrity was determined using TapeStation 2200 (Agilent). All samples analyzed had a RIN (RNA Integrity Number) number > 7.5 and were stored at −80 °C. In addition, after animal euthanization, lung tissue was collected for macroscopic examination, and no specific lesions were observed.

Microarray hybridization and sample labeling were performed according to the One-Color Microarray-Based Gene Expression Analysis - protocol version 6.9 (Agilent Technologies). Agilent RNA Spike-In mix was added to 180–220 ng of total RNA prior to the labeling reactions to monitor both labeling reactions and microarray performance, following the One-Color RNA Spike-In Kit protocol (Agilent 5188–5279). Samples were labeled using the Low Input Quick Amp Labeling kit (Agilent 5190–2306) and then hybridized to the Agilent SurePrint G3 Rat gene expression v2 8 × 60 K Microarray Kit, design ID:074036 (Agilent Technologies, Inc., CA) following the manufacturer protocol. Subsequently, total RNA was reverse transcribed to cDNA, followed by in vitro transcription and incorporation of Cy-3 fluorescent dye into the test sample. The samples were purified, dye incorporation and cRNA yield were checked with Nanodrop (NanoDrop products, Wilmington, DE, USA), then simultaneously hybridized to Agilent 8 × 60k slides for 17 h at 65 °C using Agilent’s Gene Expression Hybridization Kit (Agilent 5188–5442) according to the manufacturer instructions. The arrays were washed and scanned according to Agilent protocol using SureScan Microarray Scanner (G2600D, Agilent Technologies, Inc., CA) and one-color scanner settings. Cy3-labeling resulted in an average specific activity of 8.61 ± 3.26 pmol/μg (n = 9). All the raw data files passed the default quality control metrics that are implemented in Feature Extraction. The median ± standard error of the coefficient of variation of the within-array repeated probes calculated for the 9 samples was 4.4 ± 1.1 %. The latter indicates a within-slide technical variability of less than 5%. Data pre-processing resulted in a final set of 45,738 probes that was used for downstream analysis. The intensity data were extracted using the Feature Extraction 11.5.1.1 software (Agilent Technologies) default parameters. RNA samples obtained from all three collection tube types for each subject were run in duplicates hybridized to separate arrays on a single 8 array slide.

### 2.5 Statistical and microarray data analysis

**Fig. 1:**
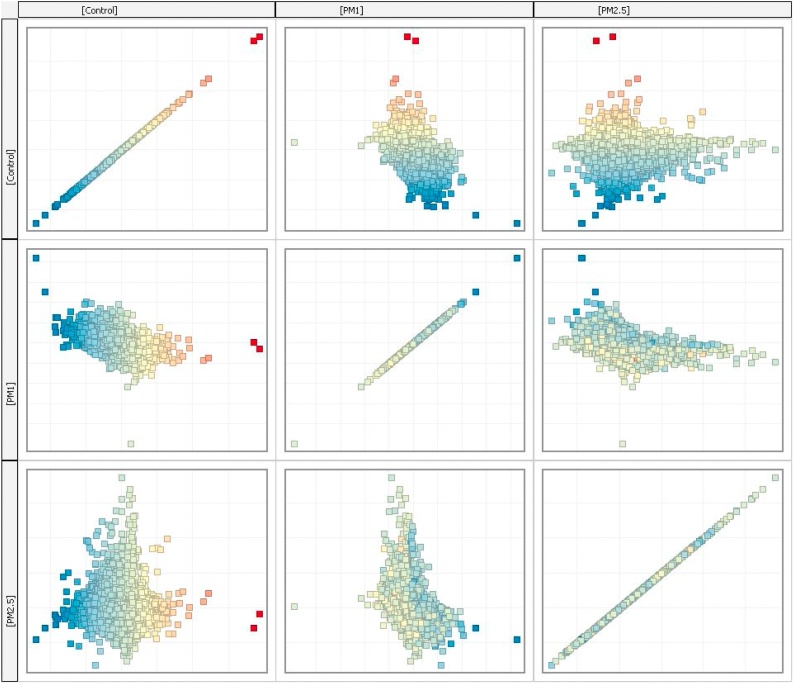
Scatter plot showing distribution of expressed probes after normalization.

Raw data were transformed to text files and were imported to Genespring software version 14.9 (Agilent) for further analysis. Since in this study there were three groups to be analyzed PM1, PM2.5 and control, pairwise comparison was performed. Raw data obtained from each sample were normalized using the Shapiro-Wilk normalization test with a p-value cut-off at 0.05. Normalized raw data were subjected to filtering by error on the 50 % of coefficient of variation (CV). Data distribution after normalization procedure is shown in Fig. 1. In every group, samples were analyzed in triplicates and the average expression of each probe in every group was used for further analysis. The different conditions that were analyzed are the following: Control-PM1, Control-PM2.5, PM2.5-PM1. In all comparisons, a moderated unpaired *t*-test was performed with a fold-change (FC) cut-off at 2.0 and a p-value cut-off at 0.05. Concerning the chemical analysis of PM filters, statistical evaluation of results was performed using SPSS (IBM SPSS Statistics version 26) and Origin 2016. A non-parametric Kruskal–Wallis test was used to identify the statistically significant differences among various groups and a p-value cut-off was set at 0.05.

## 3 Results

### 3.1 Differential gene expression on the blood transcriptome after exposure to ambient air particulate matter

**Fig. 2:**
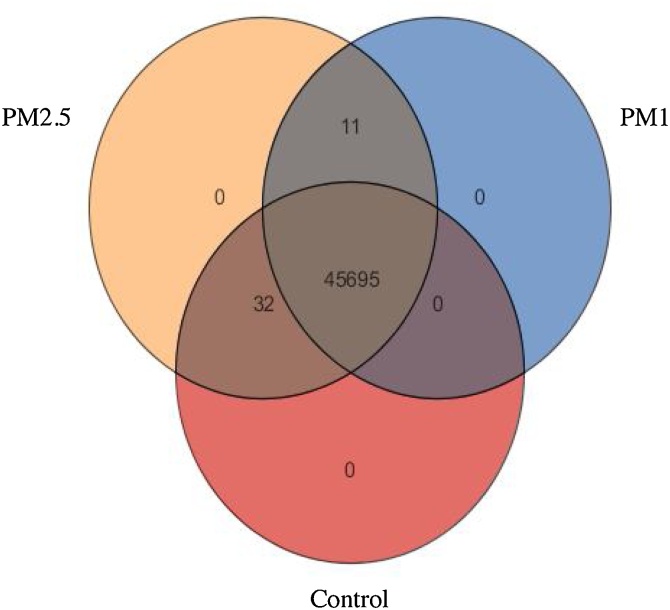
Venn diagram showing the expression of the different microarray probes. Out of 45,738 probes only 11, probes were expressed in PM2.5 and PM1 group and 32 probes were expressed in Control and PM2.5 group.

**Fig. 3:**
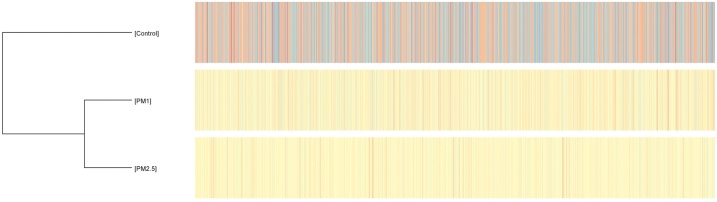
Heatmap of gene expression clustering between control (non-filtered air) group and PM1 and PM2.5 group.

To investigate the alterations in blood gene expression depending on the different air particle size inhaled by Wistar rats, a gene expression profiling analysis on blood samples was performed applying microarray probes. Two different groups of animals were exposed to PM2.5 and PM1 filtered air; a third group, designated as control was exposed to ambient air. After the background correction, 45,738 detected probes in all samples were subjected to statistical analysis on Genespring and differential pathway analysis. The Venn diagram in the following figure (Fig. 2) shows the different and common probes that were expressed in each different group, and differential gene expression in different groups is presented in a heatmap in Fig. 3. Thirty-two probes were expressed only in the control and PM2.5 group and 11 probes were expressed only in the PM1 and PM2.5 groups, whereas 45,695 probes were expressed in all study groups. In addition, after data normalization and filtering based on the coefficient of variation 18,836 probes were analyzed in pairwise conditions. Probe distribution after normality in comparison between groups is shown at the normality scatter plot (Fig. 2). General gene clustering analysis is shown in Fig. 3. Pairwise comparison of the control group compared to the PM2.5 group showed 23 differentially expressed genes (DEGs), from which 4 were significantly up-regulated and 19 were down-regulated (Table 1). The most important gene targets for further pathway analysis to study their possible biochemical role were Rasgrf1 (FC: 7.89), Plekhb1 (FC: 3.78), Trim33 (FC: -2.52), Trim65 (FC: -12.92), Car4 (FC: -3.92), S100a8 (FC: -3.52), S100a9 (FC: -2.72), Alpl (FC: -2.95), Np4 (FC: -6.97 and -11.39) and the Prok2 (FC: -16.49, and -6.97). The latter two genes were detected in different probes as precursors, a fact that can be considered as an internal validation concerning their differential expression.

**Table 1:** List of up- and down-regulated genes in control (non-filtered air) group (point of reference) compared to PM2.5 group.

Up - regulated genes				
Probe Name	Gene symbol	Gene name	p-value	Log Fold Change
A_44_P1081560	Rasgrf1	RAS protein-specific guanine nucleotide-releasing factor 1	0.00299	7.8853707
A_44_P1133465	uncharacterized LOC102551204	(LOC102551204), transcript variant X1, ncRNA	0.00311	7.952662
A_44_P1107853	Krt77	keratin 77	0.0453517	3.0366378
A_64_P028140	Plekhb1	Rattus norvegicus pleckstrin homology domain containing B1 (Plekhb1	0.025655033	3.7734509
Down - regulated genes				
A_44_P1365092	Trim33	tripartite motif-containing 33	0.04754076	−2.525949
A_44_P1151673	Cpne2	Rattus norvegicus copine 2 (Cpne2)	0.0453517	−3.6205196
A_43_P12803	Arid4b	Rattus norvegicus AT-rich interaction domain 4B (Arid4b)	0.04754076	−6.2816687
A_42_P580973	Car4	Rattus norvegicus carbonic anhydrase 4 (Car4)	0.025655033	−3.9224572
A_44_P1113190	Nek10	PREDICTED: Rattus norvegicus NIMA-related kinase 10 (Nek10), transcript variant X3	0.045351733	−3.2923589
A_44_P1157416	Trim65	Rattus norvegicus tripartite motif-containing 65 (Trim65)	0.025655033	−12.923593
A_44_P438313	Serpinb11	Rattus norvegicus serpin family B member 11 (Serpinb11)	0.045351733	−3.1229115
A_64_P141420	Serpinb1a	Rattus norvegicus serpin family B member 1A (Serpinb1a)	0.04754076	−3.541226
A_64_P062084	Birc6	Rattus norvegicus baculoviral IAP repeat-containing 6 (Birc6)	0.048876613	−2.867031
A_44_P323754	serine (or cysteine) peptidase inhibitor	clade B, member 1b	0.045351733	−14.007692
A_44_P1086470	Prok2	Rattus norvegicus prokineticin 2 (Prok2), transcript variant 1	0.042083792	−16.489689
A_44_P353618	S100a9	Rattus norvegicus S100 calcium binding protein A9 (S100a9)	0.04754076	−2.7765853
A_44_P674704	S100a8	UI-R-FS1-cqk-h-23−0-UI.s1 UI-R-FS1 Rattus norvegicus cDNA clone UI-R-FS1-cqk-h-23−0-UI 3'	0.04754076	−3.5232732
A_64_P058060	Prok2	Rattus norvegicus prokineticin 2 (Prok2), transcript variant 1, mRNA	0.030275093	−4.194672
A_43_P11684	Alpl	Rattus norvegicus alkaline phosphatase, liver/bone/kidney (Alpl)	0.04754076	−2.954144
A_44_P441181	Osr2	Rattus norvegicus odd-skipped related transciption factor 2 (Osr2)	0.04754076	−2.9556918
A_64_P032504	Np4	Rattus norvegicus defensin NP-4 precursor (Np4)	0.04754076	−6.9662805
A_44_P1107129	LOC501230	PREDICTED: Rattus norvegicus similar to nidogen 2 (LOC501230), transcript variant X1,	0.04754076	−5.1394076
A_44_P1081382	Fgd4	Rattus norvegicus FYVE, RhoGEF and PH domain containing 4 (Fgd4)	0.04754076	−2.4256036
A_44_P1113949	Np4	Rattus norvegicus defensin NP-4 precursor (Np4)	0.045351733	−11.385676
A_44_P905623	Myo7a	PREDICTED: Rattus norvegicus myosin VIIA (Myo7a), transcript variant X1	0.048876613	−6.6395516

In pairwise analysis of the PM2.5 and PM1 group, 10 significantly up-regulated genes (12 probes) for PM2.5 group were identified and no down-regulation was displayed. The identified target genes are Np4 (FC: 1.89 and 2.12), Trim65 (FC: 4.61), Alpl (FC: 3.45 and 2.41), Serpinb1a (FC: 2.22), and Serpinb11 (FC: 4.07).

Comparison of the control versus the PM1 group showed 5635 differentially regulated probes and more specifically 3069 up-regulated and 2566 down-regulated probes.

### 3.2 Gene set enrichment and pathway analysis

Due to the large amount of data produced, in order to connect the outcome to the results from previous comparisons we sought to perform a Gene Set Enrichment Analysis (GSEA) and link our results with KEGG and the WikiPathways databases. A gene set enrichment analysis was performed to identify the biochemical pathways in which the significantly differentially regulated genes at the control-PM1 pairwise comparison participate. In addition, the correlation between the target genes identified from the other pairwise analyses and the respective pathways was examined. Finally, we examined whether there are any similar genes from the same gene-families common to the other pairwise comparisons. Analyses revealed that the genes in the control group that are significantly differentially regulated compared to the PM1-group are involved in 69 different biological pathways, that include processes such as inflammatory response, cell cycle, apoptosis and pathways that may lead to carcinogenesis (Table 2). The number of differentially expressed genes in each pathway varies from 2 (Myofibroblastic activation pathway of Hepatic stellate cells) to 44 genes (MAPK signaling pathway). In interleukin signaling pathways, gene expression perturbations were observed in IL-4 (8 genes), IL-1 (9 genes), IL-7 (11 genes), IL-5 (15 genes), IL-9 (8 genes), IL-6 (20 genes), and IL-2 interleukins (20 genes). Other pathways that showed high statistical gene differentiation are the p53 pathway (15 altered genes out of 46 in the pathway), the TNF-a NF-kb signaling pathway (36 altered genes out of 175 in the pathway), the TGF-beta receptor signaling pathway (38 altered genes out of 146 in the pathway), and the T-cell receptor signaling pathway (32 altered genes out of 129 in the pathway). On the other hand, pairwise pathway analysis between the control and the PM2.5 group revealed 6 biological pathways with altered gene expression (Table 4), and comparison between the PM2.5 and the PM1 groups revealed only 2 significantly altered biological pathways; the same pathways are also included in the control-PM2.5 comparison (Table 3).

**Table 2:** Significantly differentiated genes in control group (non-filtered air) compared to PM1 group, and pathway analysis. Experiment entities column denote the number of differentially expressed genes between the two groups and the pathway entities column denote the sum of the genes involved in each biochemical pathway. P-value < 0.05.

Biological Pathway	P-value	Experiment entities	Pathway entities
1. Rn_Synthesis_and_Degradation_of_Ketone_Bodies_WP349_106980	0.015985468	3	5
2. Rn_FAS_pathway_and_Stress_induction_of_HSP_regulation_WP89_109406	0.004329809	11	37
3. Rn_IL-2_Signaling_Pathway_WP569_89844	6.8531255E-4	20	75
4. Rn_p53_signal_pathway_WP656_107003	0.011438085	8	30
5. Rn_Kit_Receptor_Signaling_Pathway_WP147_69456	0.0073107574	16	67
6. Rn_p38_MAPK_Signaling_Pathway_WP294_95743	0.04624406	8	34
7. Rn_Fatty_Acid_Beta_Oxidation_WP1307_106960	0.020708948	9	34
8. Rn_IL-6_Signaling_Pathway_WP135_83583	0.017572144	20	100
9. Rn_One_Carbon_Metabolism_WP1292_107232	0.043631077	7	27
10. Rn_EBV_LMP1_signaling_WP1278_69373	0.038883016	6	21
11. Rn_CFTR_activity_in_the_plasma_membrane_WP1488_78473	0.018428812	6	18
12. Rn_Signal_Transduction_of_S1P_Receptor_WP1312_95803	0.008289795	7	25
13. Rn_Endochondral_Ossification_WP1308_107233	0.014285385	14	61
14. Rn_Spinal_Cord_Injury_WP2433_106971	0.01612463	19	102
15. Rn_G1_to_S_cell_cycle_control_WP348_95749	0.010594943	14	66
16. Rn_Glutathione_metabolism_WP469_104998	3.3503203E-4	9	37
17. Rn_Regulation_of_Actin_Cytoskeleton_WP351_109392	0.02321428	27	146
18. Rn_Relationship_between_glutathione_and_NADPH_WP2562_106972	8.37022E-4	15	59
19. Rn_Delta-Notch_Signaling_Pathway_WP199_69380	0.005847798	18	81
20. Rn_Nuclear_factor, erythr. deriv_2,_like2_signal_pathway_WP2376_106970	0.00312188	31	161
21. Rn_EGFR1_Signaling_Pathway_WP5_69392	6.295851E-6	43	176
22. Rn_Focal_Adhesion_WP188_104997	0.009940174	35	190
23. Rn_IL-9_Signaling_Pathway_WP8_72055	0.005051836	8	24
24. Rn_Glucuronidation_WP1276_106947	0.030949743	6	20
25. Rn_G13_Signaling_Pathway_WP520_95754	0.0070167817	9	36
26. Rn_Integrin-mediated_cell_adhesion_WP74_109429	0.0331177	19	98
27. Rn_Peptide_GPCRs_WP131_71770	0.016663032	15	69
28. Rn_Apoptosis_WP1290_95785	1.1290983E-6	24	79
29. Rn_p53_pathway_WP655_89792	3.0929604E-4	15	46
30. Rn_MAPK_Cascade_WP446_95772	0.03608173	7	29
31. Rn_Type_II_interferon_signaling_(IFNG)_WP1289_86846	0.020708948	9	34
32. Rn_Apoptosis_Modulation_by_HSP70_WP487_98536	0.0012324004	8	19
33. Rn_Wnt_myofibroblastic_activ_of_Hepatic_Stellate_Cells_WP3649_89262	0.042852476	2	4
34. Rn_EPO_Receptor_Signaling_WP1284_72051	0.03608173	7	26
35. Rn_Beta_Oxidation_Meta_Pathway_WP372_70127	0.013920046	9	32
36. Rn_Wnt_Signaling_Pathway_NetPath_WP375_83028	0.03178008	20	106
37. Rn_Insulin_Signaling_WP439_96431	0.015189297	27	159
38. Rn_Angiotensin_II_signal_(acute)_thick_ascending_limbs_WP3887_107039	0.0070167817	9	29
39.Rn_NLR_Proteins_WP1294_71833	0.011185795	4	9
40. Rn_GPCRs,_Class_A_Rhodopsin-like_WP473_106994	0.014252407	40	229
41. Rn_G_Protein_Signaling_Pathways_WP73_109387	0.003612349	21	91
42. Rn_Calcium_Regulation_in_the_Cardiac_Cell_WP326_107238	0.01658603	22	142
43. Rn_IL-5_Signaling_Pathway_WP44_69358	0.01897991	15	68
44. Rn_Complement_Activation,_Classical_Pathway_WP81_107006	0.009898051	6	16
45. Rn_Cytokines_and_Inflammatory_Response_(BioCarta)_WP271_86890	0.03608173	7	26
46. Rn_Metapathway_biotransformation_WP1286_69345	0.0016079942	30	143
47. Rn_Translation_Factors_WP149_69343	0.023600718	11	47
48. Rn_Small_Ligand_GPCRs_WP161_96074	0.018428812	6	18
49. Rn_IL-1_Signaling_Pathway_WP355_79334	0.029600443	9	36
50. Rn_Cardiovascular_Signaling_WP590_109434	0.034919012	9	38
51. Rn_Estrogen_signaling_WP1279_106948	0.048943773	14	71
52. Rn_Estrogen_metabolism_WP1302_106958	0.016369272	5	14
53. Rn_TNF-alpha_NF-kB_Signaling_Pathway_WP457_69441	9.771046E-4	36	175
54. Rn_Proximal_Tubule_Transporters_WP3881_94666	0.031925526	12	54
55. Rn_MAPK_Signaling_Pathway_WP358_106982	2.3776796E-4	44	240
56. Rn_Fatty_Acid_Beta_Oxidation_2_WP105_97051	0.029020723	3	6
57. Rn_Toll-like_receptor_signaling_pathway_WP1309_72183	0.01439752	19	91
58. Rn_Androgen_Receptor_Signaling_Pathway_WP68_81858	0.0054138154	23	108
59. Rn_Cell_cycle_WP429_109404	0.007819282	19	88
60. Rn_TGF-beta_Receptor_Signaling_Pathway_WP362_69402	3.9756296E-6	38	146
61. Rn_Triacylglyceride_Synthesis_WP356_107240	0.043631077	7	27
62. Rn_IL-7_Signaling_Pathway_WP118_79681	0.014423562	11	44
63. Rn_TGF_Beta_Signaling_Pathway_WP505_109396	0.008461756	13	51
64. Rn_PKA-HCG-Glycogen_Syntase_WP2042_88326	0.020708948	9	43
65. Rn_Senescence_and_Autophagy_WP1305_86844	0.03178008	20	104
66. Rn_PI3K-AKT-NFKB_pathway_WP1491_89860	0.0012647143	21	86
67. Rn_B_Cell_Receptor_Signaling_Pathway_WP285_89912	1.3381344E-6	41	155
68. Rn_T_Cell_Receptor_Signaling_Pathway_WP352_69416	6.849953E-5	32	129
69. Rn_IL-4_Signaling_Pathway_WP182_69413	0.001262184	16	58

**Table 3:** Pathway analysis of significantly differentiated genes in control group (non-filtered air) compared to PM2.5 group, and between PM2.5 and PM1 group. Experiment entities column denote the number of differentially expressed genes between each pairwise analysis and the pathway entities column denote the sum of the genes involved in each biochemical pathway. P-value < 0.05.

Control – PM2.5			
Biological Pathway	P-value	Experiment entities	Pathway entities
1. Rn_p38_MAPK_Signaling_Pathway_WP294_95743	0.019649707	1	34
2. Rn_Endochondral_Ossification_WP1308_107233	0.035450514	1	61
3. Rn_Thick_Ascending_Limb_Transporters_WP3882_106985	0.011359166	1	19
4. Rn_TNF-alpha_NF-kB_Signaling_Pathway_WP457_69441	0.09733374	1	175
5. Rn_Proximal_Tubule_Transporters_WP3881_94666	0.031960495	1	54
**PM2.5 – PM1**			
Biological Pathway	P-value	Experiment entities	Pathway entities
1. Rn_Endochondral_Ossification_WP1308_107233	0.017173307	1	61
2. Rn_TNF-alpha_NF kB_Signaling_Pathway_WP457_69441	0.04795735	1	175

### 3.3 Chemical analysis of PM filters

**Fig. 4:**
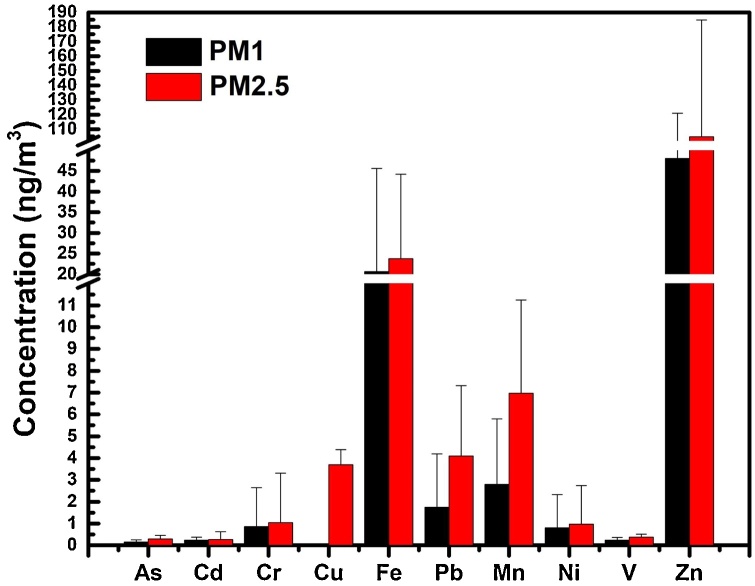
Comparison between water-soluble trace element concentrations in PM1 and PM2.5 samples from the Kalamaria ambient air monitoring station.

The mean concentration of PM1 and PM2.5 during the sampling period was 17.7 ± 9.0 μg/m^3^ and 29.1 ± 16.1 μg/m^3^, respectively (Table 4, Table 5). The levels of PM2.5 exceeded the threshold of 25 μg/m^3^ proposed by the 2008/50/EC guide values for 25 days. The maximum concentration for PM2.5 was 91.6 μg/m^3^ observed on February 11, 2020. PM-component analysis showed high concentrations of Fe, Pb, Mn and Zn for both particle size analysis, while high concentration of Cu was observed in PM2.5 filters (Fig. 4). Size–dependent solubility was detected for Cu; Cu solubility was higher in the PM2.5 size fraction compared to PM1 – solubility was negligible in that particle size fraction. The sampling site in Kalamaria has lower traffic, so crustal elements are expected to have higher concentrations. The water-soluble fractions identified in the atmosphere of Kalamaria site were found to represent 0.36 % and 0.52 % of the total mass of PM1 and PM2.5, respectively. To investigate whether the differences in mean concentrations of the water-soluble concentrations between PM1 and PM2.5 fractions were significant, Kruskal–Wallis test was performed. The latter test indicated significant differences among the two fractions for the concentrations of V (p = 0.003), Zn (p = 0.008), As (p = 0.028), Pb (p = 0.036), and Mn (p = 0.002), whereas no significant differences were found for Ni (p = 0.125), Cd (p = 0.235), Cr (p = 0.130), and Fe (p = 0.574). The ratios of PM_1_/PM_2.5_, compared between water soluble fractions for Cd, Cr, Fe and Ni were higher than 0.50, leading to the conclusion that these elements are predominantly distributed over the PM1 fraction.

**Table 4:** Average, minimum and maximum concentrations of PM1 and water-soluble fractions of the measured elements for the city of Thessaloniki. *ND: Not Detected.

Elements	Units	Mean	SD	MIN	MAX
As	ng/m^3^	0.15	0.10	0.004	0.40
Cd	ng/m^3^	0.24	0.135	0.005	0.464
Cr	ng/m^3^	0.86	1.78	0.03	6.46
Cu	ng/m^3^	ND	ND	ND	ND
Fe	ng/m^3^	20.66	24.93	0.54	82.14
Pb	ng/m^3^	1.74	2.45	0.00	9.65
Mn	ng/m^3^	2.80	2.99	0.78	12.50
Ni	ng/m^3^	0.80	1.52	0.04	4.74
V	ng/m^3^	0.24	0.12	0.10	0.58
Zn	ng/m^3^	47.98	72.96	1.09	269.01
PM_1_	μg/m^3^	17.7	9.0	6.8	42.9

**Table 5:** Average, minimum and maximum concentrations of PM2.5 and water-soluble fractions of the measured elements for the city of Thessaloniki.

Elements	Units	Mean	SD	MIN	MAX
As	ng/m^3^	0.29	0.17	0.06	0.65
Cd	ng/m^3^	0.27	0.35	0.03	1.08
Cr	ng/m^3^	1.05	2.26	0.02	8.73
Cu	ng/m^3^	3.69	0.68	3.21	4.18
Fe	ng/m^3^	23.8	20.44	0.94	48.26
Pb	ng/m^3^	4.1	3.22	0.06	10.32
Mn	ng/m^3^	6.97	4.27	0.96	15.05
Ni	ng/m^3^	0.96	1.78	0.06	6.69
V	ng/m^3^	0.38	0.13	0.16	0.67
Zn	ng/m^3^	104.8	80.01	5.46	277.2
PM_2.5_	μg/m^3^	29.1	16.1	6.8	91.6

## 4 Discussion

For the first time according to the literature, the exposure effect of ambient particles PM2.5 and PM1 on gene expression level was studied in real-life exposure conditions using Wistar rats. The experimental design regarding animal exposure followed an adapted version of an applied protocol that used mice [19]. The Samara et al. study focused only on PM10 exposure and analyzed only the macroscopic observations in lungs after exposure. Another significant difference is that in the current study the control group received non-filtered atmospheric air, instead of HEPA-filtered clean air. The reason we chose this control is that we sought to identify the genes that are differentially regulated in animals exposed to atmospheric air as a mixture and compare the outcome to the PM2.5 and PM1 animal groups, thus, focusing in particular on the effect of particle size.

During the last years several teams performed in vivo research on the effects of PM2.5 exposure on animals [18,[20], [21], [22], [23]]. In these studies, particulate matter is administered to animals as mixtures in several routes such as intraorally, intranasally, intratracheally or using a nebulizer and thus, not following the natural dose of exposure. The real-life exposure conditions we tried to achieve in this study offers the advantage of obtaining high-quality results after exposure from the natural route and ambient concentrations of air pollutants, 24 h/7 days without trying to extrapolate our conclusions from the lab to the field.

In general, current evidence suggests that the smaller the particle size is, the more pronounced the health effects are, due to the fact that PM1 particles are more likely to reach into deeper compartments of the respiratory system, thus transferring several pathogens and toxins originate from anthropogenic emissions [24,25]. To date there is no conclusive evidence regarding health effects of PM1, due to the fact that is not regulated and routinely monitored around the world; nevertheless, a study performed in 26 cities in China showed that both exposures to ambient PM1 and PM2.5 were significantly associated with increased emergency hospital visits and most of the health effects of PM2.5 came from PM1 [26].

Surprisingly, in our study, gene expression comparison of the control group (atmospheric air) to the PM1 group showed 5635 differentially expressed probes, whereas the comparison of the control group to the PM2.5 group showed only 23 differentially expressed probes. For this reason, we chose to select gene-targets for further analysis from the PM2.5 group. Due to the fact that there were three groups to be analyzed and compared, only genes that were significantly differentially expressed (p-value: <0.05) and showed a fold change of >2.0 were considered in the statistical analysis.

Our overall results show the possible significant impact due to differential gene expression regulation of the smaller particle size on health effects compared to non-filtered atmospheric air. Another question raised out of the results is what differential regulation of certain genes in PM1 group means if the same genes are not up-regulated in the control group since both groups are exposed to PM1, one independently and the other in the mixture. The answer is that it is not known yet how the different size particles interact with each other within a mixture compared to the effect each size category shows on its own.

Further detailed experimentation is needed to evaluate the impact of the particle size ranging between 2.5 and 1 μm and ultrafine particles (<0.1 μm). The enhanced adverse health effects of smaller particles like PM1 is likely due to higher pulmonary deposition efficiency, easier vascular penetration, larger surface area, and the concentration of more toxic components (higher specific concentration due to the absorptive capacity of the larger surface area).

One of the genes that were significantly up-regulated in the control group inhaling non-filtered air compared to the other two groups is RASGRF1 (FC: 7.9). RASGRF1 is a guanine nucleotide exchange factor, which promotes the release of GDP from inactive Ras and stabilizes the apoprotein; its hypermethylated status was found to be a potential risk factor for colorectal cancer, in experiments performed in rats [27]. Thus, further experiments are needed to evaluate its expression in lung tissue and unravel the mechanism of action to RAS cell signaling pathways that it is known to play a role in biochemical pathways linked to carcinogenesis [28,29].

TRIM65 (FC: -12.92) is an important gene that was significantly down-regulated in the control group. That gene acts as a negative regulator of miRNA activity, regulating miRNA-driven suppression of mRNA translation by targeting TNRC6 (trinucleotide repeat containing six) proteins for ubiquitination and degradation [30]. Thus, the biochemical pathways in which this gene is involved should be investigated further with more targeted experiments and in other tissue substrates such as lungs, oral and nasal cavity and lymph nodes of the upper respiratory system. Transcriptomic analysis has shown also down-regulation of TRIM33 (FC: -2.52) or ubiquitin protein ligase, which is a transcriptional intermediary factor that in humans is expressed in high amounts in the lungs and in the brain, and was found to restrain HIV-1 infection intracellularly by targeting viral integrase for proteasomal degradation. In mice it was shown that TRIM33, promotes the proinflammatory function of Th17 cells by inducing IL-17 and suppressing IL-10 expression [31,32]. At this point, it should be noted that pairwise comparison of the PM2.5 vs. the PM1 group showed up-regulation of the TRIM65 gene (FC: 4.61) in the PM2.5 group. The latter result indicates that it would be interesting to explore further the role of fine particles in the size range between 2.5 and 1 μm on TRIM65 regulation and the factors that contribute to and alter its expression.

Another gene-target that showed significant down-regulation in the control group (FC: -11.39), while it was up-regulated in the PM2.5 (FC: 2.01) compared to PM1 group is the NP4 gene, which is the precursor of defensin-4. That protein is very important for defense against bacterial and viral pathogens in rats; in general, defensins play an participate in antiviral mechanisms and pathogenesis and is present at the surface of the mucosa [33]. Thus, it is a very important indication that in the non-filtered control group NP4 is down-regulated indicating that several mechanisms that are protective against pathogen invasion are disactivated, whereas analysis of the data from the smaller size groups (PM2.5 and PM1) showed up-regulation of NP4; this indicates that the burden induced by the smaller size particles compared to the overall ambient air mixture is not enough to suppress those mechanisms. Calprotectin is a protein that is encoded by S100A8 (FC: -3.52), and S100A9 (FC: -2.72) genes, which are both down-regulated in the control group. The protein is a Ca^+2^ binding protein of the S100 protein family, and is expressed constitutively in monocytes and in neutrophils, while it plays a major role in inflammation by stimulating leukocyte recruitment and inducing cytokine secretion [34]. This finding is another indication of the down-regulation or possibly the disactivation of anti-inflammatory mechanisms due to exposure in urban or industrial atmospheric air, and thus, paving the way for pathogens to invade easier into the organism through the mucosal surfaces of the respiratory epithelium or the gastrointestinal tract.

An important result is the up-regulation of the ALPL gene (FC: -2.95 in the control group). Epidemiological evidence has shown that mutations of that gene are linked to hypophosphatasia, which is a rare genetic disorder characterized by abnormal development of bones and teeth, in Chinese and Spanish populations [35,36].

Finally, one of the most significant findings in this study is the statistically significant down-regulation of the PROK2 (prokinetisin-2) gene. The latter is a member of the prokineticin gene family and the respective signaling pathways are implicated in several important physiological functions, including gastrointestinal smooth muscle contraction, circadian rhythm regulation, neurogenesis, angiogenesis, pain perception, mood regulation, and reproduction [37]. In addition, dysregulation of prokineticin signaling has been observed in a variety of diseases, such as cancer, ischemia, and neurodegeneration, in which prokineticin signaling seems to be a promising therapeutic target. Recently, specific phenotypes of PROKR2 and PROK2 knockout mice, have been identified as causative genes for idiopathic hypogonadotropic hypogonadism, a developmental disorder characterized by impaired development of gonadotropin-releasing hormone neurons and infertility [38]. Mutations of PROK2 has also been suggested to be the causative agent for Kallmann syndrome in children [39]. Since, PROK2 is involved in many biological procedures and is linked to the serious disorders highlighted above it is important to identify the environmental factors that alter the regulation of this gene and search for possible mutations due to environmental exposure. It is a very good starting point for the development of adverse outcome pathways of each specific target gene linked to specific environmental factors.

For this reason, in this study, in parallel with the in vivo “real-life” experiments, PM1 and PM2.5 ambient concentrations were measured daily and showed a mean concentration of 177 ± 9,0 μg/m^3^ and 291 ± 161 μg/m^3^ respectively. High concentrations of Zn and Pb are typically attributed to the abrasive effect of vehicles and they can indicate a possible source from the mechanical parts of vehicles such as brake abrasion [40,41]. In addition, the water-soluble Fe and Mn concentrations were higher compared to those reported for a city center station in Patras (38.2466 °N, 21.7346 °E), and these two trace elements have been shown to be related to soil resuspension [42,43]. Significant differences between PM1 and PM2.5 fractions were found for V, Zn, As, P and Mn. According to Strickland et al. [44] when the concentration of the water-soluble metals Cr, Cu, Fe, Mn, Ni and V exceeds 12 ng/m^3^ the emergency visits due to child asthma showed a statistically significant increase. In the current study this value was exceeded for both PM1 (254 ng/m^3^) and PM2,5 (368 ng/m^3^) samples suggesting the possible health risk due to metal concentration levels in the studied areas. A recent study has shown that Mn, Cd, Cu, Pb, Ni and Cr(VI) displayed a significant risk for human health associated with inhalation exposure to water-soluble heavy metals of indoor PM2.5 [45]. Moreover, the same study showed that water soluble heavy metal concentrations displayed higher inhalation risk compared to labile fractions.

In the present study, gene candidate biomarkers have been identified. The next step would be the linkage to specific metals and the respective health outcome, in order to develop novel AOPs due to environmental exposure. Nevertheless, heavy metals are not the only substances included in particle mixtures, and in future research polycyclic aromatic hydrocarbons (PAHs) should be included in the context of novel AOP development after “real-life” exposure. It has already been shown that the relationship of lung cancer risk and PAHs is dependent on the particle size, and that the smaller the particle size, the higher is the accumulation in deeper parts of the lower respiratory tract and the PAH burden [46]. Moreover, vehicle-emitted PAHs has been shown to inhibit proliferation of specific T-cell subtypes, and negatively regulate immune cell subtypes of the blood compartment [47].

Differentially expressed genes (DEGs) were assigned to specific molecular pathways of the KEGG library and WikiPathways in order to link specific genes to cell signaling pathways. Pathway analysis of differentially expressed genes found in the control-PM1 group comparison revealed a series of biological procedures including innate immunity inflammation, cell cycle, apoptosis, mitogen-activated protein kinases (MAPK) and oxidative stress. Currently, there is ongoing research on the PM-induced inflammatory response caused by atmospheric toxicity factors, and a recent study developed a cell-line system that revealed a relationship between induction of inflammatory response and the atmospheric endotoxin level [48]. Experiments with alveolar macrophages and lung epithelial cells have shown that exposure to PM2.5 induces oxidative stress through TNF-a and IL-6 activation, and apoptosis through the p53, c-Myc and p21 signaling pathway [49,50]. In addition, transcriptome analysis of the skin barrier of rats showed increased cholesterol synthesis and skin damage induced by PM2.5 [51]. Still, with an even smaller particle size such as ultrafine particles (UFPs), it was shown immediate detection of particles in blood after inhalation and preservation in lungs for up to 6 h after instillation, causing severe inflammatory responses and macrophage chemotaxis by activating IL-4, IL-5, IL-6, IL-10 and IL-13 [[52], [53], [54]]. Our results showed severe activation of the interleukin cascade of events between control and PM1 group and the respective pathways displayed many differentially expressed genes in IL-4 (8 genes), IL-1 (9 genes), IL-7 (11 genes), IL-5 (15 genes), IL-9 (8 genes), IL-6 (20 genes), and IL-2 interleukins (20 genes). Our results correlate also with the activation of p53 (15 genes), TNF-a (36 genes) and in addition strong activation was shown in T-cell (32 genes), B-cell (41 genes), and Toll-like (19 genes) receptor pathways. A recent study showed that Toll-like receptor 4/MyD88 pathway activation was one of two major immune responses in acute lung inflammation in mice after intratracheally instillation of PM2.5 (Wang et al., 2017).

In this research, a novel protocol to study “real-life” exposure of different size ambient air particles on Wistar rats was applied and furthermore, to our knowledge, it is the first time that whole blood mRNA transcriptome has been mapped and compared after exposure to real time experimental conditions. Despite the fact that this is the first study of a series of experiments that will result in the development of adverse outcome pathways (AOPs) caused by PM2.5 and PM1 exposure, our research had certain limitations. Since the biochemical perturbations caused by environmental pollution can trigger a cascade of events in the cell biology and metabolism we sought firstly to “map” the transcriptome on the gene expression level and select gene candidates after strong statistical power analysis. Thus, in order to circumvent potentially confounding factors such as oestrus cycles and the impact of hormones on physiological functions, only male rats were included in this study. A recent study that included rats has examined the transcription factors that contribute to sex bias evolutionary changes and it was revealed that around 3000 genes were sex biased regulated in gene expression [55]. Another factor that should be taken into account in further research is the mapping of the transcriptome. In this study, we analyzed the blood transcriptome on gene expression (mRNA) level using microarray probes, meaning that the analysis has not considered the transcription factors such as miRNAs and DNA methylation traits that have been regulated due to particle exposure and contribute to gene expression. A previous study using rats has demonstrated that a specific miRNA is possibly involved in hepatocellular carcinoma metastasis by regulating positively osteoblast differentiation from progenitor cells [56]. Thus, the continuation of the current research would be the identification of miRNAs that regulate the differentially expressed genes after exposure to PM2.5 and PM1.

Moreover, future experiments should also focus on tissue specific analysis in lungs, and in the nasal and oral cavity which are the portals of entry for particle exposure. Gene expression analysis in the latter tissue compartments will link the differentially regulated genes with the blood transcriptome and heavy metals involved in biochemical alterations, leading to the identification of novel AOPs and to validation of specific biomarkers of exposure. Recent studies on rats has shown the long-term effect after exposure to specific chemical mixtures and xenobiotics, where they found genotoxic and cytotoxic effects in a tissue- and a dose-dependent manner [57,58]. Selected genes should be examined epigenetically in order to identify the transcription factors such as microRNAs or small interfering RNAs (siRNAs) that participate in the regulation of the significantly regulated genes.

In summary, “real-life” exposure of Wistar rats showed significant alterations in gene expression through whole genome microarray analysis. These alterations may be related with high concentrations of heavy metals present in particle mixtures. Furthermore, on the basis of our results we can conclude the following:

1) the smaller the size of the inhaled particles, the more gene alterations are triggered, as in our results differentially regulated genes between control-PM1 comparison were 200 times more than the control-PM2.5 comparison;

2) specific target-genes were selected as candidate biomarkers of effect after exposure to filtered or non-filtered atmospheric air for further research in order to develop novel AOPs leading to carcinogenicity and other pathogenic conditions and;

3) a “real-life” exposure model using Wistar-rats was evaluated for further use by the scientific community in future research.

## Author statement

Conception and design of study: Ilias S. Frydas, Athanasios Salifoglou, Dimosthenis A. Sarigiannis

Acquisition of data: Ilias S. Frydas, Kermenidou Marianthi, Olga Tsave

Analysis and/or interpretation of data: Ilias S. Frydas, Kermenidou Marianthi

Drafting the manuscript: Ilias S. Frydas

Revising the manuscript critically for important intellectual content: Dimosthenis A. Sarigiannis

Approval of the version of the manuscript to be published (the names of all authors must be listed): Ilias S. Frydas, Kermenidou Marianthi, Olga Tsave, Athanasios Salifoglou, Dimosthenis A. Sarigiannis

## Declaration of Competing Interest

The authors declare that they have no known competing financial interests or personal relationships that could have appeared to influence the work reported in this paper.

## References

[1] Kloog I., Ridgway B., Koutrakis P., Coull B.A., Schwartz J.D. (2013). Long- and short-term exposure to PM2.5 and mortality using novel exposure models. Epidemiology.

[2] Manisalidis I., Stavropoulou E., Stavropoulos A., Bezirtzoglou E. (2020). Environmental and Health Impacts of Air Pollution: A Review.

[3] Pope C.A., Burnett R.T., Thun M.J. (2002). Lung Cancer, cardiopulmonary mortality, and long-term exposure to fine particulate air pollution. JAMA.

[4] Filep Á., Fodor G.H., Kun-Szabó F. (2016). Exposure to urban PM1 in rats: development of bronchial inflammation and airway hyperresponsiveness. Respir. Res..

[5] Brüggemann E., Gerwig H., Gnauk T., Müller K., Herrmann H. (2009). Influence of seasons, air mass origin and day of the week on size-segregated chemical composition of aerosol particles at a kerbside. Atmos. Environ..

[6] Kelly F.J., Fussell J.C. (2020). Size, source and chemical composition as determinants of toxicity attributable to ambient particulate matter. Atmos. Environ..

[7] Beule A.G. (2010). Physiology and pathophysiology of respiratory mucosa of the nose and the paranasal sinuses. GMS Curr. Top. Otorhinolaryngol. Head Neck Surg..

[8] Sanders N.N., De Smedt S.C., Van Rompaey E., Simoens P., De Baets F., Demeester J. (2000). Cystic fibrosis sputum: a barrier to the transport of nanospheres. Am. J. Respir. Crit. Care Med..

[9] Atuma C., Strugala V., Allen A., Holm L. (2001). The adherent gastrointestinal mucus gel layer: thickness and physical state in vivo. American J. physiology. Gastrointestinal liver physiology.

[10] Ensign L.M., Tang B.C., Wang Y.Y., Tse T.A., Hoen T., Cone R., Hanes J. (2020). Mucus-penetrating nanoparticles for vaginal drug delivery protect against herpes simplex virus. Sci. Transl. Med..

[11] Ljubimov A.V.J.Y., Kleinman M.T., Karabalin N.M., Inoue S., Konda B., Gangalum P., Markman J.L., Ljubimov A.V., Black K.L. (2013). Gene expression changes in rat brain after short and long exposures to particulate matter in Los Angeles basin air: comparison with human brain tumors. Exp. Toxicol. Pathol..

[12] Liu L., Urch B., Szyszkowicz M. (2017). Influence of exposure to coarse, fine and ultrafine urban particulate matter and their biological constituents on neural biomarkers in a randomized controlled crossover study. Environ. Int..

[13] Lorelei de Jesus A., Rahman M., Mazaheri M., Thompson H., Knibbs L.D., Jeong C., Evans G., Nei W., Ding A., Qiao L., Li L., Portin H., Niemi J.V., Timonen H., Luoma K., Petäjä T., Kulmala M., Kowalski M., Peters A., Cyrys J., Ferrero L., Manigrasso M., Avino P., Buonano G., Reche C., Querol X., Beddows D., Harrison R.M., Sowlat M.H., Sioutas C., Morawska L. (2019). Ultrafine particles and PM2.5 in the air of cities around the world: are they representative of each other?. Environ. Int..

[14] Franck U., Odeh S., Wiedensohler A., Wehner B., Herbarth O. (2011). The effect of particle size on cardiovascular disorders – the smaller the worse. Sci. Total Environ..

[15] Chung M., Wang D.D., Rizzo A.M., Gachette D., Delnord M., Parambi R., Brugge D. (2020). “Association of PNC, BC, and PM2.5 measured at a central monitoring site with blood pressure in a predominantly near highway population. Int. J. Environ. Res. Public Health.

[16] Atkinson W.R., Fuller W.G., Anderson R.H., Harrison M.R., Armstrong M.B. (2010). Urban ambient particle metrics and health: a time-series analysis. Epidemiology.

[17] Amato F., Moreno T., Pandolfi M., Querol X., Alastuey A., Delgado A. (2010). Concentrations, sources and geochemistry of airborne particulate matter at a major European airport. J. Environ. Monit..

[18] Farina F., Sancini G., Longhin E., Mantecca P., Camatini M., Palestini P. (2013). Milan PM1 induces adverse effects on mice lungs and cardiovascular system. Biomed Res. Int..

[19] Samara C., Kouras A., Kaidoglou K., Emmanouil-Nikoloussi E., Simou C., Bousnaki M., Kelessis A. (2015). Ultrastructural alterations in the mouse lung caused by real-life ambient PM10 at urban traffic sites. Sci. Total Environ..

[20] Xiao C., Li S., Zhou W., Shang D., Zhao S., Zhu X., Chen K., Wang R. (2013). The effect of air pollutants on the microecology of the respiratory tract of rats. Environ. Toxicol. Pharmacol..

[21] Ljubimova J.Y., Braubach O., Patil R. (2018). Coarse particulate matter (PM2.5–10) in Los Angeles Basin air induces expression of inflammation and cancer biomarkers in rat brains. Sci. Rep..

[22] Ouyang Y., Xu Z., Fan E. (2018). Changes in gene expression in chronic allergy mouse model exposed to natural environmental PM2.5-rich ambient air pollution. Sci. Rep..

[23] Shen Y., Zhang Z., Hu D. (2018). The airway inflammation induced by nasal inoculation of PM2.5 and the treatment of bacterial lysates in rats. Sci. Rep..

[24] Liu L., Breitner S., Schneider A. (2013). Size-fractioned particulate air pollution and cardiovascular emergency room visits in Beijing. China. Environ Res.

[25] Meng X., Ma Y., Chen R., Zhou Z., Chen B., Kan H. (2013). Size-fractionated particle number concentrations and daily mortality in a Chinese city. Environ. Health Perspect..

[26] Chen G., Li S., Zhang Y. (2017). Effects of ambient PM1 air pollution on daily emergency hospital visits in China: an epidemiological study. Lancet Planet. Health.

[27] Chen H., Xu Z., Yang B., Zhou X., Kong H. (2018). RASGRF1 Hypermethylation, a Putative Biomarker of Colorectal Cancer. Ann. Clin. Lab. Sci..

[28] Gurung A.B., Bhattacharjee A. (2015). Significance of ras-signaling in cancer and strategies for its control. Oncol. Hematol. Rev..

[29] Wen Z., Jiang R., Huang Y. (2019). Inhibition of lung cancer cells and Ras/Raf/MEK/ERK signal transduction by ectonucleoside triphosphate phosphohydrolase-7 (ENTPD7). Respir. Res..

[30] Li S., Wang L., Fu B., Berman A.M., Diallo A., Dorf M.E. (2014). TRIM65 controls TNRC6 stability and miRNA function. PNAS.

[31] Tanaka S., Jiang Y., Martinez G.J. (2018). Trim33 mediates the proinflammatory function of Th17 cells. J. Exp. Med..

[32] Ali H., Mano M., Braga L. (2019). Cellular TRIM33 restrains HIV-1 infection by targeting viral integrase for proteasomal degradation. Nat. Commun..

[33] Holly M.K., Diaz K., Smith J.G. (2017). Defensins in viral infection and pathogenesis. Annu. Rev. Virol..

[34] Wang S., Song R., Wang Z., Jing Z., Wang S., Ma J. (2018). S100A8/A9 in inflammation. Front. Immunol..

[35] Xu L., Pang Q., Jiang Y. (2018). Four novel mutations in the *ALPL* gene in Chinese patients with odonto, childhood, and adult hypophosphatasia. Biosci. Rep..

[36] García-Fontana C., Villa-Suárez J.M., Andújar-Vera F. (2019). Epidemiological, clinical and genetic study of Hypophosphatasia in a spanish population: identification of two novel mutations in the alpl gene. Sci. Rep..

[37] Zhao Y., Wu J., Wang X., Jia H., Chen D.N., Li J.D. (2019). Prokineticins and their G protein-coupled receptors in health and disease. Prog. Mol. Biol. Transl. Sci..

[38] Matsumoto S., Yamazaki C., Masumoto K.H. (2006). Abnormal development of the olfactory bulb and reproductive system in mice lacking prokineticin receptor PKR2. Proc Natl Acad Sci U S A.

[39] Mitchell A.L., Dwyer A., Pitteloud N., Quinton R. (2011). Genetic basis and variable phenotypic expression of Kallmann syndrome: towards a unifying theory. Trends Endocrinol. Metab..

[40] Athanasopoulou E., Tombrou M., Russell A.G., Karanasiou A., Eleftheriadis K., Dandou A. (2010). Implementation of road and soil dust emission parameterizations in the aerosol model CAMx: applications over the greater Athens urban area affected by natural sources. J. Geophys. Res. Atmos..

[41] Chernyshev V.V., Zakharenko A.M., Ugay S.M., Hien T.T., Hai L.H., Olesik S.M., Kholodov A.S., Zubko E., Kokkinakis M., Burykina T.I., Stratidakis A.K., Mezhuev Y.O., Sarigiannis D.A., Tsatsakis A., KS Golokhvast (2018). Morphological and chemical composition of particulate matter in buses exhaust. Toxicol. Rep..

[42] Manousakas M., Papaefthymiou H., Eleftheriadis K., Katsanou K. (2014). Determination of water-soluble and insoluble elements in PM2.5 by ICP-MS. Sci. Total Environ..

[43] Manousakas M., Diapouli E., Papaefthymiou H., Migliori A., Karydas A.G., Padilla-Alvarez R., Bogovac M., Kaiser R.B., Jaksic M., Bogdanovic-Radovic I., Eleftheriadis K. (2015). Source apportionment by PMF on elemental concentrations obtained by PIXE analysis of PM10 samples collected at the vicinity of lignite power plants and mines in Megalopolis, Greece. Nucl. Instrum. Methods Phys. Res. B.

[44] Strickland M.J., Darrow L.A., Klein M., Flanders W.D., Sarnat J.A., Waller L.A., Sarnat S.E., Mulholland J.A., Tolbert P.E. (2010). Short-term associations between ambient air pollutants and pediatric asthma emergency department visits. Am. J. Respir. Crit. Care Med..

[45] Kogianni E., Kouras A., Samara C. (2020). Indoor concentrations of PM_2.5_ and associated water-soluble and labile heavy metal fractions in workplaces: implications for inhalation health risk assessment. Environ. Sci. Pollut. Res. Int..

[46] Sarigiannis D.A., Karakitsios S.P., Zikopoulos D., Nikolaki S., Kermenidou M. (2015). Lung cancer risk from PAHs emitted from biomass combustion. Environ. Res..

[47] Zakharenko A.M., Engin A.B., Chernyshev V.V., Chaika V.V., Ugay S.M., Rezaee R., Karimi G., Drozd V.A., Nikitina A.V., Solomennik S.F., Kudryavkina O.R. (2017). Basophil mediated pro-allergic inflammation in vehicle-emitted particles exposure. Environ. Res..

[48] Yamagishi N., Yamaguchi T., Kuga T., Taniguchi M., Khan M.S., Matsumoto T., Deguchi Y., Nagaoka H., Wakabayashi K., Watanabe T. (2020). Development of a system for the detection of the inflammatory response induced by airborne fine particulate matter in rat tracheal epithelial cells. Toxicol. Rep..

[49] Becker S., Mundandhara S., Devlin R.B., Madden M. (2005). Regulation of cytokine production in human alveolar macrophages and airway epithelial cell in response to ambient air pollution particles: further mechanistic studies. Toxicol. Appl. Pharmacol..

[50] Huang Q., Zhang J., Peng S., Tian M., Chen J., Shen H. (2014). Effects of water soluble PM2.5 extracts exposure on human lung epithelial cells (A549): a proteomic study. J. Appl. Toxicol..

[51] Liao Z., Nie J., Sun P. (2020). The impact of particulate matter (PM2. 5) on skin barrier revealed by transcriptome analysis: focusing on cholesterol metabolism. Toxicol. Rep..

[52] Mills N.L., Amin N., Robinson S.D. (2006). Do inhaled carbon nanoparticles translocate directly into the circulation in humans?. Am. J. Respir. Crit. Care Med..

[53] de Haar C., Haasing I., Bol M., Bleumink R., Pieters R. (2006). Ultrafine but not fine particle matter causes airway inflammation and allergic airway sensitization to co-administered antigen in mice. Clin. Exp. Allergy.

[54] Li N., Wang M., Bramble L.A. (2009). The adjuvant effect of ambient particulate matter is closely reflected by the particulate oxidant potential. Environ. Health Perspect..

[55] Naqvi S., Godfrey A.K., Hughes J.F., Goodheart M.L., Mitchell R.N., Page D.C. (2019). Conservation, acquisition, and functional impact of sex-biased gene expression in mammals. Science.

[56] Shi Y., Huang A. (2015). Effects of sorafenib on lung metastasis in rats with hepatocellular carcinoma: the role of microRNAs. Tumor Biol..

[57] Fountoucidou P., Veskoukis A.S., Kerasioti E., Docea A.O., Taitzoglou I.A., Liesivuori J., Tsatsakis A., Kouretas D. (2019). A mixture of routinely encountered xenobiotics induces both redox adaptations and perturbations in blood and tissues of rats after a long-term low-dose exposure regimen: The time and dose issue. Toxicol. Lett..

[58] Tsatsakis A., Docea A.O., Constantin C., Calina D., Zlatian O., Nikolouzakis T.K., Stivaktakis P.D., Kalogeraki A., Liesivuori J., Tzanakakis G., Neagu M. (2019). Genotoxic, cytotoxic, and cytopathological effects in rats exposed for 18 months to a mixture of 13 chemicals in doses below NOAEL levels. Toxicol. Lett..

[59] U.S. EPA. Air Quality Criteria for Particulate Matter (Final Report, April 1996). U.S. Environmental Protection Agency, Washington, D.C., EPA 600/P-95/001.

